# Probabilistic Pocket Druggability Prediction *via* One-Class Learning

**DOI:** 10.3389/fphar.2022.870479

**Published:** 2022-06-29

**Authors:** Riccardo Aguti, Erika Gardini, Martina Bertazzo, Sergio Decherchi, Andrea Cavalli

**Affiliations:** ^1^ Computational and Chemical Biology, Fondazione Istituto Italiano di Tecnologia, Genoa, Italy; ^2^ Department of Pharmacy and Biotechnology, University of Bologna, Bologna, Italy

**Keywords:** druggability prediction, drug design, machine learning, unsupervised methods, one-class classification, import vector domain description, conceptron

## Abstract

The choice of target pocket is a key step in a drug discovery campaign. This step can be supported by *in silico* druggability prediction. In the literature, druggability prediction is often approached as a two-class classification task that distinguishes between druggable and non-druggable (or less druggable) pockets (or voxels). Apart from obvious cases, however, the non-druggable class is conceptually ambiguous. This is because any pocket (or target) is only non-druggable until a drug is found for it. It is therefore more appropriate to adopt a one-class approach, which uses only unambiguous information, namely, druggable pockets. Here, we propose using the import vector domain description (IVDD) algorithm to support this task. IVDD is a one-class probabilistic kernel machine that we previously introduced. To feed the algorithm, we use customized DrugPred descriptors computed *via* NanoShaper. Our results demonstrate the feasibility and effectiveness of the approach. In particular, we can remove or mitigate biases chiefly due to the labeling.

## 1 Introduction

Drug discovery is a time-consuming and complex task (Nicolaou, 2014). It requires a multistep pipeline from biological understanding to fine-tuning of the lead candidate (for small molecules), often *via* computational means (Csermely et al., 2013; Jamali et al., 2016). In the past 20 years, computation has significantly contributed to many drug discovery steps *via* physics-based simulation, machine learning modeling, and the combination of the two (Decherchi and Cavalli, 2020b; Decherchi et al., 2021).

In particular, computational modeling can help find a putatively druggable target and hence a pocket that may accept a small molecule. A protein of interest is considered druggable when a drug has been found to inhibit it. However, some authors consider ligandability to be a more appropriate term for the propensity of the target/protein to accept drug-like molecules, irrespective of the more complex pharmacokinetic and pharmacodynamic mechanisms implied by the term druggability (Edfeldt et al., 2011). Here, we use the term druggable pocket to indicate a region of a protein with a high probability of accepting a small molecule. The reliable *in silico* identification of potentially druggable pockets is important for drug discovery. Finding new druggable hot spots can be particularly relevant when searching for an allosteric binder and to boost selectivity. Selectivity, in turn, is particularly important when designing chemical entities like PROTACs (Shimokawa et al., 2017; Qi et al., 2021), even more relevant than optimizing the affinity of the warhead itself. While researchers often know about the orthosteric pocket of a specific protein, it requires geometric and chemical insights to detect alternate druggable pockets, making it a much more complex task. Effective tools are therefore required to support the computational medicinal chemist in detecting and ranking new pockets in order to design highly selective drugs.

The literature contains many reports on the computational estimation of druggability (Agoni et al., 2020). The available tools for this task include standalone software [e.g., P2Rank (Krivák and Hoksza, 2018)] and web servers [e.g., PockDrug (Hussein et al., 2015)]. Prediction often involves defining geometric and chemical features to support machine learning techniques (Xie et al., 2009) [e.g., DrugPred (Krasowski et al., 2011)]. Alternatively, more recent deep learning methodologies often use 3D grids (voxels) of physicochemical fields. Indeed, there are several methods for predicting the probability of a pocket’s druggability. DoGSiteScorer (Volkamer et al., 2012b) is an algorithm that detects pockets and estimates druggability by considering global and local pocket proprieties. It uses support vector machines to build a predictive model. PRANK (Krivák and Hoksza, 2015) uses decision trees and random forests to re-rank/re-score the pockets predicted by other software, such as ConCavity (Capra et al., 2009) and Fpocket (Le Guilloux et al., 2009). PRANK could help improve the performance of existing prediction methods; it aims to predict the ligandability of a specific point near the surface of the pocket. TRAPP is a powerful method for analyzing molecular dynamics trajectories. It was recently endowed with druggability assessment capabilities, extending its analysis to an entire ensemble of structures (Yuan et al., 2020).

Druggability can also be assessed with pharmacophores (Desaphy et al., 2012) by using either very simple geometric considerations (e.g., Cavity (Yuan et al., 2013)) or fully fledged deep learning approaches. There are many such deep learning approaches, which often leverage convolutional neural networks coupled to 3D grids. In Zhang et al. (2020), the authors used both the pocket and the ligand with DenseNet architecture. In contrast, Pu et al. (2019) used convolutional neural networks specialized for nucleotide and heme-binding sites, again starting from 3D grids. InDeep (Mallet et al., 2021) is another contribution based on a convolutional architecture. Here, the focus is on characterizing protein–protein interfaces (PPI) to allow designing of PPI disruptors. The capabilities of convolutional neural networks were boosted by pocket segmentation in Aggarwal et al. (2021). This work and others [e.g., Stepniewska-Dziubinska et al. (2020)] demonstrated that both prediction and other activities, such as segmentation, are beneficial, so one can devise a more complex framework than a pure predictor. Along these lines, PUResNet (Kandel et al., 2021) uses an interesting deep residual (skip connections) decoder/encoder architecture derived from the U-net concept. This work presented both an architecture and a cleanup procedure for the training set. This class of deep methods is very accurate but lacks native interpretability.

From the protein dataset perspective, some datasets used in published works are suitable benchmarks. They are often used to train and test machine learning protocols, thus creating a shared base. For instance, in Hajduk et al. (2005), the authors created an online dataset containing 72 unique protein-binding sites. The authors in Schmidtke and Barril (2010) published two datasets: a large but redundant dataset (DD, with 1,070 structures) and a non-redundant subset (70 binding sites).

Here, we address the problem of druggability estimation from the perspective of bias mitigation. The *a priori* dichotomy between druggable and less druggable (or non-druggable) pockets technically supports machine learning classifiers. Conceptually, however, it is questionable to use or define a non-druggable class. Indeed, apart from trivial cases (e.g., very small pockets), it is at best ambiguous to define such class. Defining a pocket as non-druggable (or less druggable) automatically creates a bias in the learned model, which may hamper the detection of a potentially useful pocket. Hence, we argue that druggability estimation should be approached as a one-class unsupervised learning task, not a classification one. This is because a classification task would inevitably create arbitrary user-dependent biases in the definition of the non-druggable (or less druggable) class. Starting from this observation, we devised a protocol that uses the import vector domain description method (a probabilistic one-class non-linear learner) to learn a hypersphere (a generalized minimum enclosing ball), which contains druggable pockets (Decherchi and Rocchia, 2016; Decherchi and Cavalli, 2020a). That is, only the definition of a druggable pocket is required during training, avoiding the creation of bias in the definition of the non-druggable class. To support the learner, we used a NanoShaper-based implementation of DrugPred (Krasowski et al., 2011) descriptors with minor modifications (the entrance area computed by NanoShaper is used as an additional descriptor). We employed the dataset in Krasowski et al. (2011) because it is widely used and explicitly defines a less druggable set of pockets. Furthermore, we defined a diversified new dataset of 100 protein targets to further validate the method. This dataset is a subset of the Potential Drug Target Database (PDTD (Gao et al., 2008)). Our results demonstrate the effectiveness of the approach. In the following, Section 2 describes the method workflow, Section 3 shows the results of the experiments, and Section 4 introduces possible future developments and reports the final conclusions.

## 2 Methods

**FIGURE 1:**
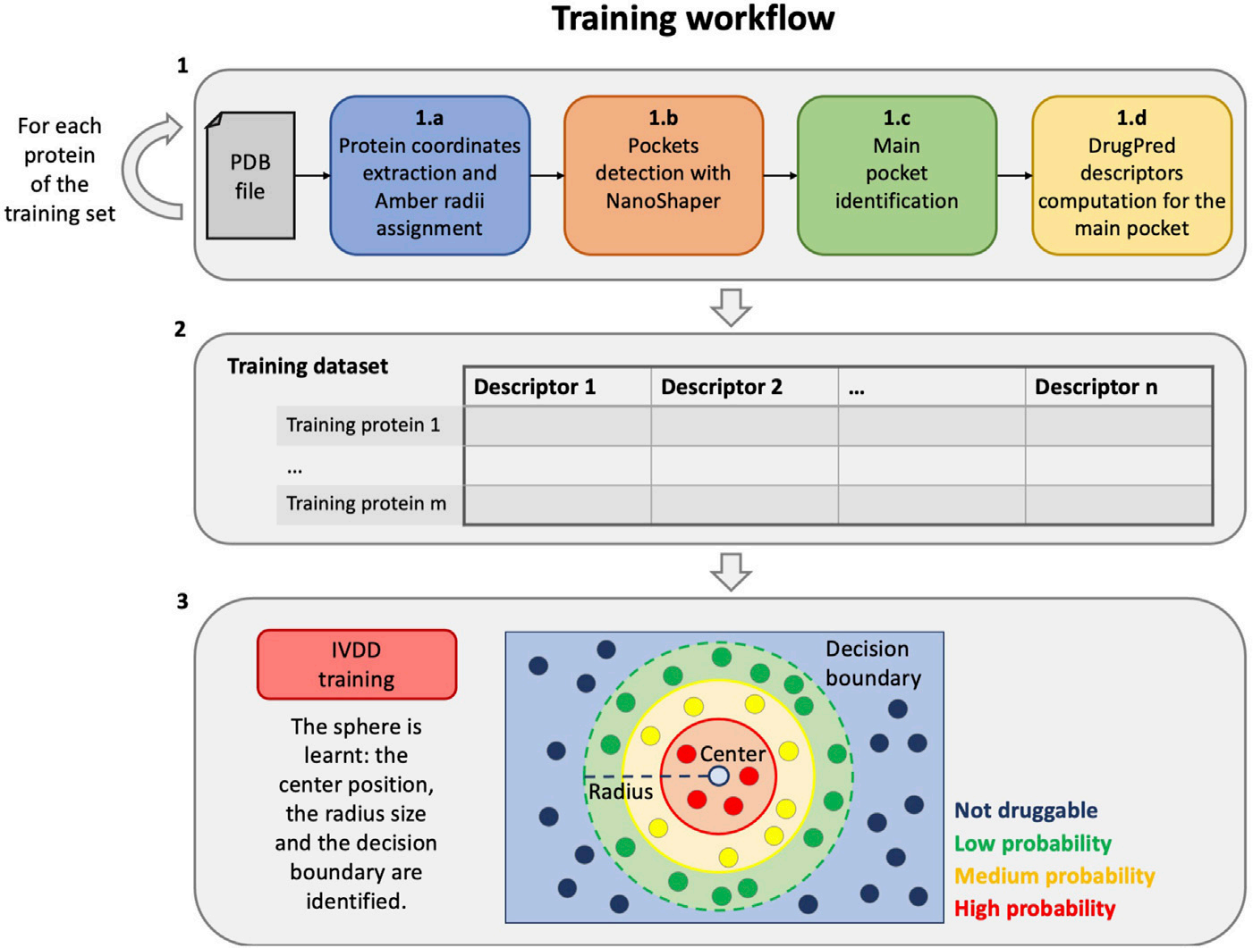
Training workflow. From the creation of the training dataset to the training phase of the IVDD method.

In this section, we have described the proposed workflow for druggability prediction. For clarity, we have separated the training workflow from the testing (the operative phase) one. The training phase is a step that is required to estimate (learn) the model and comprises three main steps (see Figure 1):

1 First, we compute descriptors for the proteins of the training set, in particular, for each protein, as follows:
  a) the protein part is filtered from the input PDB, and the radii of the Amber99SB-ildn force field are assigned to it;
  b) the PDB file is thus converted to a .xyzr file and then passed to NanoShaper to detect all the pockets;
  c) a main druggable pocket is identified (one for each training protein);
  d) the geometric and chemical descriptors of the pocket are computed.
2 All the information from the previous step is aggregated in order to form the training dataset, which is therefore composed by the descriptors of each main druggable pocket of the training targets.
3 Finally, the training dataset is used to train the import vector domain description (IVDD) machine learning method. In this phase, a sphere is learned and allows to assign a probability value to each pocket and consequently to distinguish druggable (probability ≥0.5 ) and non-druggable pockets (probability <0.5 ).

**FIGURE 2:**
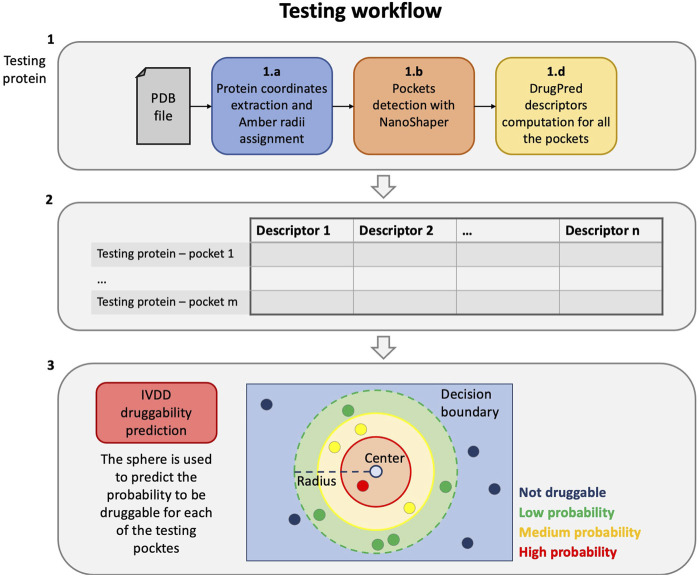
Testing workflow. From the protein PDB file to the druggability prediction with the trained IVDD.

On the other hand, the testing/operative protocol, that is, when the model is used for predictions only, comprises three main steps (see Figure 2):

1 First, we compute the descriptors for the current target protein, as follows:
  a) the protein part is filtered from the input PDB and the radii of the Amber99SB-ildn force field are assigned to it;
  b) the PDB file is thus converted to a .xyzr file and then passed to NanoShaper to detect all the pockets;
  c) the geometric and chemical descriptors of the pockets are computed.
2 All the information from the previous step is aggregated obtaining a single file comprised of the descriptors of each pocket of the current target.
3 Finally, the previously estimated hypersphere is used to predict the probability of each of the newly detected pockets. The pockets with the highest probability are most likely to be druggable.

In the following sections we provide more details regarding the abovementioned steps. In particular, Section 2.1 describes steps 1b and 1c of the pipeline, Section 2.2 provides information regarding the descriptors building step (1d), and finally, Section 2.3 explains the IVDD method mentioned in step 3.

### 2.1 NanoShaper Pockets Detection and Main Pocket Identification

The detection of all the available pockets is instrumental for estimating the druggability of each pocket in the protein of interest. For this step, we used the NanoShaper tool (Decherchi and Rocchia, 2013; Decherchi et al., 2018) to efficiently deliver the set of pockets on a protein. NanoShaper was chosen as it accurately estimates the molecular surface (Wilson and Krasny (2021)); the detected pockets are triangulated with the same technique used for molecular surface triangulation, hence providing smooth triangulated meshes.

The detected pockets are saved as mesh files in MSMS or in the .off format, and they can be easily parsed to support the subsequent descriptors building step. NanoShaper also provides volume, surface area, and a list of the constituining atoms for all the internal cavities and pockets identified for the given molecular system. These are identified and computed *via* an intuitive approach, which involves a volumetric difference of the regions of the space between system’s solvent-excluded surfaces (SESs), with two probe radii, dubbed a large probe (with radius *R*) and a small one (with radius *r*) (Decherchi et al., 2018). The probe sizes encode the expectation onto the shape of the pockets. High *R* values allow the identification of shallow pockets, whereas high *r* values will smooth inner surface gaps. Default values are 3.0 Å and 1.4 Å for the large and small probes, respectively. The large radius is based on empirical evidence and the small radius mimics the water molecule. Here, we used the default value of the small radius but fine-tuned the large radius to a value of 3.5 Å. With respect to the default value of 3.0 Å, we found that this value allows a better detection of slightly more shallow pockets (a larger surface size of pocket entrance).

To create the training dataset, we needed an automated method to detect the orthosteric/main pocket, where the ligand is located, and discriminate it from the others (NanoShaper delivers several pockets). Because the orthosteric pocket is well-identified in the analyzed PDB, we used the surrounding atoms of the ligand. In detail, we used the Jaccard index on the atom indices to easily detect the ortostheric pocket; the Jaccard index of atoms is an accurate proxy of the discretized volume overlap, often found in druggability predictors. We defined the orthosteric pocket as the pocket detected by NanoShaper with the maximal Jaccard index with respect to the reference indices. This is easily achieved by localizing the atom indices around target’s natural substrate (or drug). The Jaccard index is defined as follows:

$$J\left(O,P_{i}\right)=\frac{\left|O\cap P_{i}\right|}{\left|O\cup P_{i}\right|},$$ (1)

where O is the indices set for the orthosteric site and *P*_*i*_ is the set of detected atom indices in the *i*th pocket. The Jaccard index is hence a natural measure of the quality of the detected pocket with respect to ligand’s envelope. One can note that the Jaccard index can be decomposed into two components, which account for the degree of overimposition of the pocket and reference ligand volume in two different ways. The first component is the normalized intersection component *J*_*int*_:

$$J_{int}\left(O,P_{i}\right)=\frac{\left|O\cap P_{i}\right|}{\left|O\right|},$$ (2)

and the second one is the normalized union component *J*_*or*_:

$$J_{or}\left(O,P_{i}\right)=\frac{\left|O\right|}{\left|O\cup P_{i}\right|}.$$ (3)

They both belong to the interval (0,1). They account, respectively, for the ability to detect all the reference atoms (*J*_*int*_) and the tightness of detection (*J*_*or*_). Both properties are desirable and consistently lead to the Jaccard index upon multiplication. To fairly evaluate the results, we considered these metrics together with classification accuracy.

### 2.2 Descriptors Building

To characterize each pocket identified by NanoShaper, we used the descriptors defined by Krasowski et al. (2011) together with the entrance area provided by NanoShaper (Table 1).

**TABLE 1:** Descriptors of the datasets. The incidence is calculated for every amino acid X.

Descriptor	Abbr
Binding site volume	vol
Total surface area	area_b
Entrance area	area_e
Binding site compactness	cness
Relative hydrogen-bond donor surface area	dsa_r
Hydrogen-bond donor surface area	dsa_t
Relative hydrogen-bond acceptor surface area	asa_r
Hydrogen-bond acceptor surface area	asa_t
Relative hydrophobic surface area	hsa_r
Hydrophobic surface area	hsa_t
Relative occurrence of polar amino acids	paa
Relative occurrence of non-polar amino acids	haa
Relative occurrence of multifunctional amino acids	maa
Relative occurrence of charged amino acids	caa
Relative polar surface area (dsa_r + asa_r)	psa_r
incidence of amino acid X in the binding site relative to the surface	in_X

Binding site properties describing size, shape, polarity, and amino acid composition were calculated using NanoShaper output files as input to the descriptors builder. In particular, to compute volume (vol), total surface area (area_b), and entrance area (area_e) (which describes the area of the pocket mouth), we directly used the estimations provided by NanoShaper. To calculate the other descriptors, we started from the NanoShaper output files describing the atoms and meshes of each pocket. The hydrogen-bond donor and acceptor properties of each pocket were calculated by considering the surface area surrounding all the polar atoms (dsa_t and asa_t). Based on these descriptors, the hydrophobic surface area (hsa_t) is defined as the difference between the total surface area and the sum of the hydrogen-bond donor and acceptor surface areas. Moreover, relative amplitude of the hydrogen-bond donor and acceptor surface areas (dsa_r and asa_r) and the hydrophobic surface area (hsa_r) were computed by dividing each descriptor by the total surface area of the binding site. Finally, the relative polar surface area (psa_r) is defined as the sum between the relative hydrogen-bond donor and acceptor surface areas. To characterize the shape of different cavities, we used the compactness descriptor, defined by Krasowski et al. (2011):

$$cness=\frac{4\pi {\left(\sqrt[{3}]{\frac{vol}{\frac{4}{3}\pi }}\right)}^{2}}{area\text{\_}b}.$$ (4)

According to this equation, the closer the compactness is to 1, the more spherical is the pocket. The remaining descriptors, relating to amino acid composition, were calculated by considering the occurrence of different classes of amino acids grouped by their overall physicochemical properties. In particular, all the amino acids were grouped into the following classes:

• Apolar: Ala, Gly, Val, Ile, Leu, Met, Phe, and Pro.
• Polar: Thr, Lys, Arg, Glu, Asp, Gln, Asn, and Ser.
• Charged: Lys, Arg, His, Asp, and Glu.
• Multifunctional: Trp, Tyr, His, and Cys.

To define the relative occurrence of hydrophic amino acids (haa), polar amino acids (paa), charged amino acids (caa), and multifunctional amino acids (maa), we computed the fraction of each group of amino acids with respect to the total number of amino acids comprising each cavity. Finally, we reported the incidence of each amino acid of type (in_X) as descriptors, defined as the sum of all the surface areas surrounding the amino acid X.

### 2.3 Druggability Estimation *via* IVDD One-Class Learning

**FIGURE 3:**
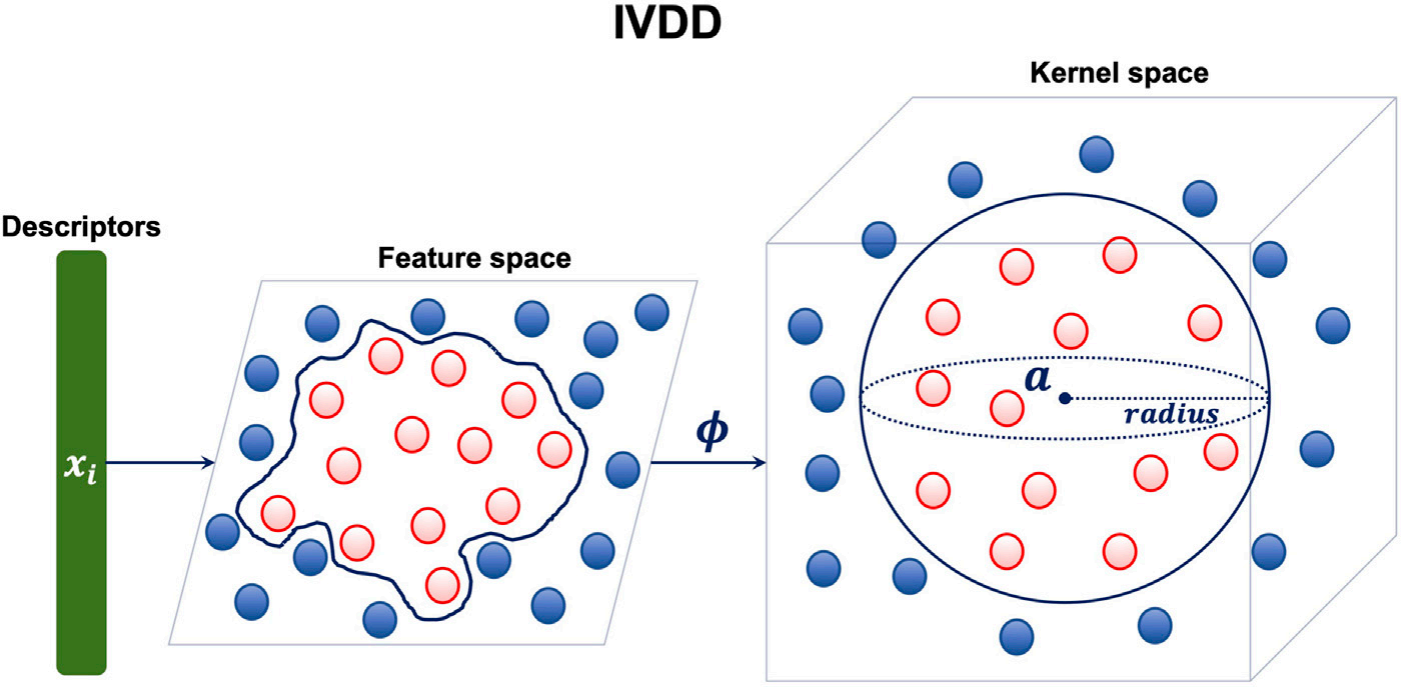
IVDD method: each sample (druggable pocket) is a single point in a *d*-dimensional space (here *d* =35, which is the number of descriptors). The hypersphere is created in a kernel space. The mapping between the feature space and the kernel space is given by the function *ϕ*.

As anticipated, we used a one-class approach, that is, we require and consider for the training phase only the samples in the class from which we want to learn the concept. The aim is to learn the concept of a *druggable* pocket. This requires only samples (pockets) that are known to be druggable. To perform this step, we used the one-class learner dubbed import vector domain description (Decherchi and Rocchia (2016)). The import vector domain description method tries to embed the available training samples into an enclosing hypersphere. This sphere does not belong to the original input space but rather resides in a, possibly infinite dimensional, kernel space. This approach allows us to wrap the data in arbitrarily complex enclosing surfaces because the hypersphere in kernel space corresponds to a not necessarily spherical enclosing surface in the original space (see Figure 3).

This makes the method very flexible. Moreover, the enclosing surface is endowed with a probabilistic model, which assigns the probability of belonging (or not) to the enclosing sphere.

The aim of the training procedure of IVDD is to find a sphere configuration (center position and radius size) that best minimizes the cost function (see later). The cost function tries to maintain as much as possible the samples inside the sphere while at the same time keeping under control the radius size, possibly letting some training samples outside the sphere. One is eventually searching for a compact representation of the space spanned by the samples. We will call [*π*_*low*_, *π*_*high*_] the range of acceptance of the fraction of training examples inside the sphere. It can be shown that the optimal sphere (the solution of the minimization problem) is unique, as the problem is convex. Once the final sphere configuration is found it determines predictions during the operative phase. The non-druggable nature of a pocket is just an interpretation over the probability values; strictly speaking, one-class learning just describes the adherence of a sample (a pocket) to a concept (druggability). If a crisp classification is needed, the probability threshold of 0.5 can be used. Samples outside the sphere (decision boundary) are predicted as non-druggable (with a corresponding probability lower than 0.5), while samples inside the sphere are predicted as druggable (with a corresponding probability higher than 0.5). Clearly, the inner and most central pockets are estimated to have the highest probabilities of being druggable. Indeed, this probability is high at the core of the sphere and decreases toward the edges.

At a mathematical level, the training phase of the IVDD method is characterized by the following minimization problem:

$$\underset{\Gamma ,a}{\min}\Gamma^{2}-\hat{C}\overset{n}{\underset{i=1}{\sum }}\log\left(p_{i}\right),$$ (5)

where Γ is the square of the radius of the hypersphere, constant $\hat{C}=C/n$ represents the trade-off between the radius size and the error minimization, and *p*_*i*_ is the probability defined by a logistic model:

$$p_{i}=\frac{1}{1+\exp\left(\beta f_{i}\right)},$$ (6)

where *β* is a fixed coefficient and *f*_*i*_ is the decision function defined as follows:

$$f_{i}=d^{2}\left(\Phi \left(x_{i}\right),a\right)-\Gamma ,$$ (7)

where *d* ^2^ (Φ(**x**_*i*_), **a**) is the distance function and **a** is the center of the hypersphere. The cost function in Eq. 5 is optimized *via* an efficient learning algorithm that can be ascribed to a class of sequential minimal optimization (SMO) algorithms (Zeng et al., 2008). The introduced probability model is used to probe the druggability of each pocket. We refer the reader to Decherchi and Rocchia (2016) for further details.

## 3 Results

### 3.1 Datasets

In this work, we used two different datasets to run the experiments. In both cases, we generated two versions of the dataset: with and without hydrogen atoms. The first dataset is the NRDLD dataset, presented in Krasowski et al. (2011). It is the largest publicly accessible non-redundant dataset for model building and validation of structure-based druggability methods. The dataset comprises 115 structures (protein-binding sites), including 71 druggable and 44 less druggable (which becomes 42 after the analysis in Krasowski et al. (2011)). For each binding site, 35 different descriptors are calculated, as described in section 2.2 and summarized in Table 1.

In addition to the NRDLD dataset, we created another dataset comprising the binding sites of 100 different proteins. Those targets are taken from the PDTD (Potential Drug Target Database) (Gao et al., 2008), a free online collection of 1,100 3D structures of proteins. The targets in our 100-protein dataset include enzymes, receptors, antibodies, signaling proteins, and lipid-binding proteins. We thus obtained 5,692 and 4,807 binding sites without and with hydrogen atoms, respectively. Of these, 100 are orthosteric (one for each target). For each structure, we selected the pocket that hosts the drug or substrate. We avoided selecting pockets that host cofactors. We defined these pockets as orthosteric (or main) throughout the text (because the drug is co-crystallized in the orthosteric site in most cases). As for the NRDLD dataset, we calculated previously defined descriptors for each binding site (see Table 1).

For more information on the targets of the NRDLD and the PDTD datasets, see Supplementary Material Sections S1, S2.

### 3.2 Model Training

We trained IVDD considering the descriptors of *n* = 70 druggable structures in the NRDLD dataset. The *1nvj* structure was excluded since it represents a small oligonucleotide and we only considered proteins to calculate the descriptors. The following parameters were adopted: kernel used is RBF with *σ* = max_*ij*_ (*d*_*ij*_)/log(*n*) (where *d*_*ij*_ is the distance between the *i-th* and the *j-th* sample); value of *C* is initialized as 0.5, the value of *β* is set as 25, while the range of accepted inner samples is set to [*π*_*low*_, *π*_*high*_] = [0.8, 0.9]. The values of [*π*_*low*_, *π*_*high*_] may vary according to the reliability of the training dataset. In this case, we preferred a conservative approach, with 80–90% of samples included inside the sphere and the remaining peripheral 20–10% as outliers, in order to avoid overfitting. The learning phase is stopped when the range of inner samples is hit. Each time the training is repeated, the *C* is increased/reduced by 0.01 (increased if the percentage of samples inside the sphere is lower than the desired range, reduced otherwise). In our case, the training procedure ended with 90*%* samples inside the sphere and a final *C* value of 0.1 for the solution without hydrogen atoms and with 90*%* of samples inside the sphere and a final *C* value of 0.12 for the solution with hydrogen atoms.

**FIGURE 4:**
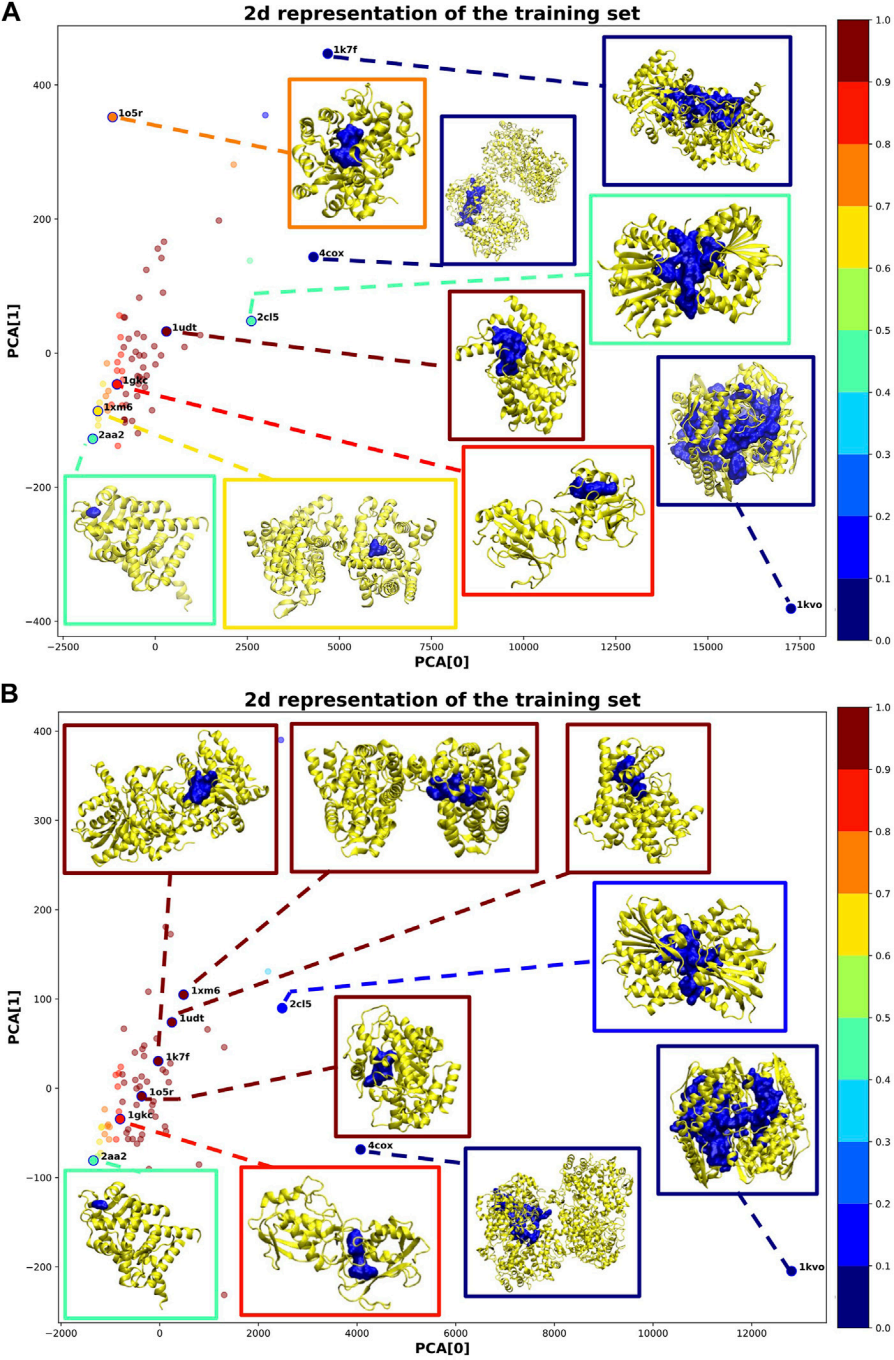
2D representation of the training samples *via* PCA dimensionality reduction. Each point corresponds to a training sample (protein-binding site). The color of each point corresponds to the probability assigned by IVDD (graded according to the color map on the right). For some training samples, the corresponding 3D structure is shown. **(A)** is without hydrogen atoms, whereas in **(B)** hydrogen atoms were added.

Figure 4 shows a 2D representation of the training set obtained by reducing the dimensionality *via* a principal component analysis (PCA) (Jolliffe, 1986). For some samples, we additionally plotted the corresponding 3D structure. In both cases, most of the training samples coherently obtained a high probability of druggability (dark red points in Figure 4). This outcome is obtained because we imposed the solution to include at least 80*%* of the training samples inside the sphere.

Considering the solution without hydrogen atoms (see Figure 4A), the sample *1udt* has the highest probability and is the sample nearest to the center of the sphere. In this structure, the pocket identified by NanoShaper is very compact and well-defined. IVDD performs the best in cases where the pocket closely surrounds the ligand bound in it. The samples outside the sphere (corresponding to 10*%* of the samples) obtained low probability scores. These scores are explainable by looking at the pocket shape. Structures such as *1kvo*, *4cox*, and *1k7f* do not look like well-defined pockets but rather like a fusion of more than one pocket. This leads to descriptors that are quite distant from those that the algorithm is learning as the druggable reference. As a consequence, those structures are scored as outliers. This highlights that *ex post* segmentation can be a powerful preprocessing tool before the machine learning step. Nevertheless, IVDD can cope with this situation by excluding or marginalizing percolating pockets. It is possible to identify another case where NanoShaper did not correctly identify the orthosteric pocket (i.e., *2aa2*). Here, the pocket is very shallow and the bound ligand is not deeply buried. The identified pocket is much smaller than it should be, leading to a low probability. This effect is expected because NanoShaper can only detect shallow pockets *via* a proper tuning of the big probe, whereas the selected value is expected to work mainly for deep buried prototypical pockets.

The solution with hydrogen atoms (see Figure 4B) identifies the sample *1xm6* as having the highest probability. In contrast to the solution without hydrogen atoms, its structure is now more compact around the ligand with a greater *J*_*int*_. Since the presence of hydrogen atoms better defined the orthosteric pocket, NanoShaper improved its accuracy, leading to a high IVDD probability. This happened similarly for *1k7f*, where the channel that led to a big pocket was closed by the presence of hydrogen atoms. In this specific case, NanoShaper identified the orthosteric pocket with a Jaccard index three times better than the solution without hydrogen atoms. Although the solution with hydrogen atoms solved some NanoShaper errors (wide percolation), pockets such as *1kvo*, *4cox*, and *2aa2* remained more or less unchanged, with very big or shallow structures. The option to use hydrogen atoms (or not) is partially data-dependent and is further studied in NRDLD and new datasets.

### 3.3 Experiment on the NRDLD Dataset

**FIGURE 5:**
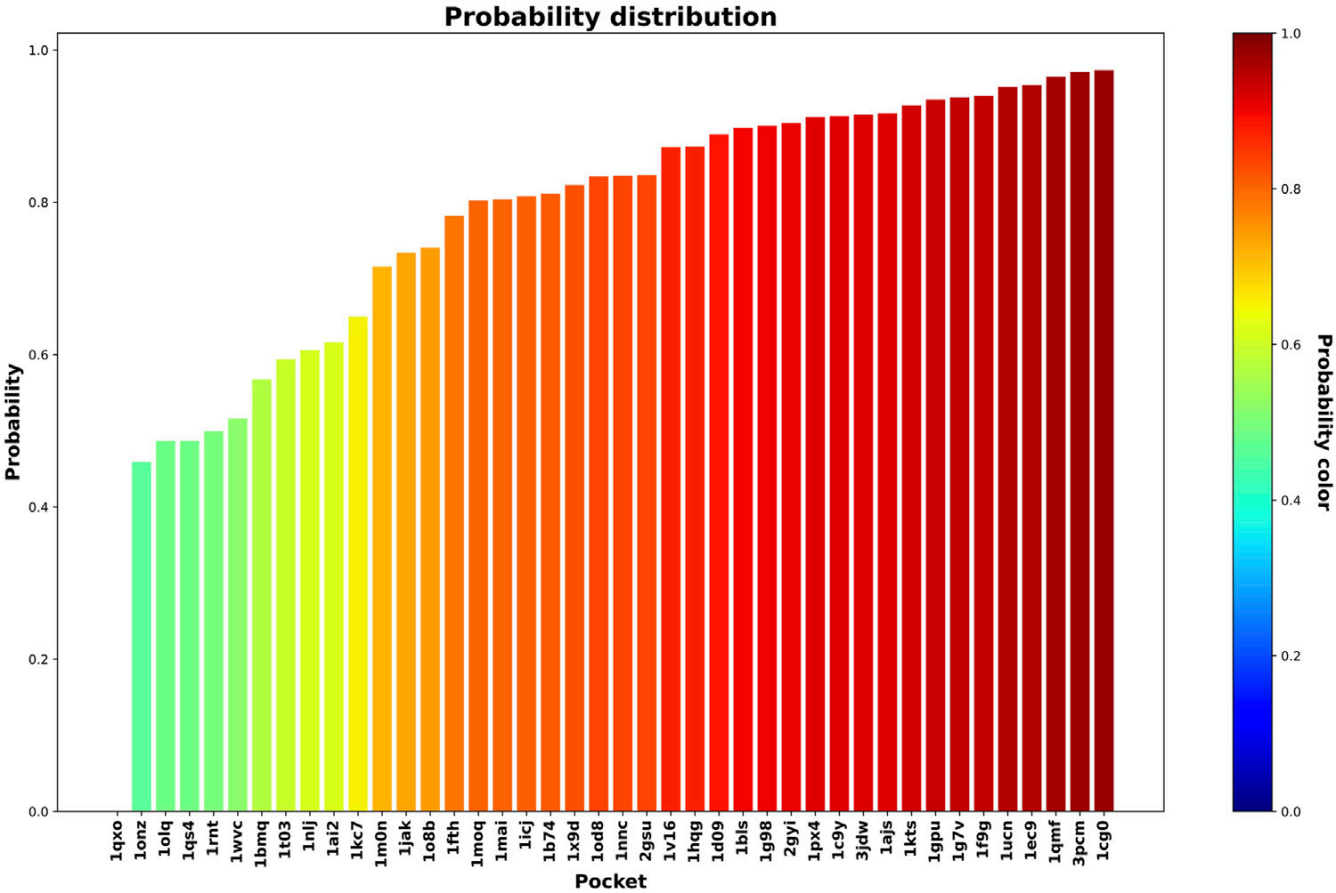
Druggability prediction for the less druggable subset of the NRDLD dataset without adding hydrogen atoms. For each protein-binding site (the *x*-axis), we predicted its druggability probability (the *y-*axis and color of the bar).

**FIGURE 6:**
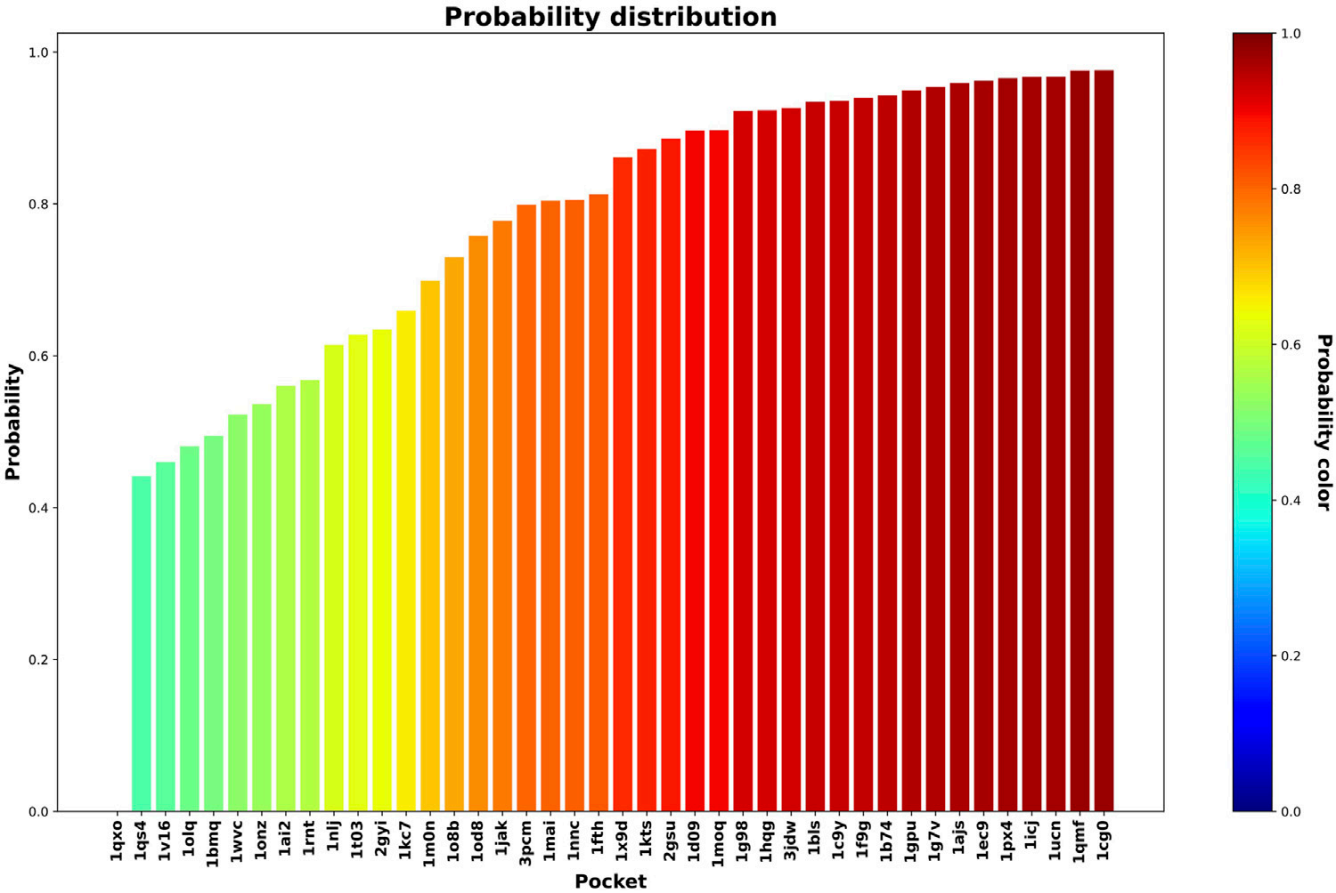
Druggability prediction for the less druggable subset of the NRDLD dataset with the addition of hydrogen atoms. For each protein binding site (the *x*-axis), we predicted its druggability probability (the *y*-axis and color of the bar).

In this step, we used the 42 less druggable structures described in Krasowski et al. (2011) in order to test the previously trained model and perform druggability prediction. Figures 5 and 6 show the probability assigned to each structure by the IVDD method for the solutions without and with hydrogen atoms, respectively.

Generally speaking, the following results are relatively similar. The resulting trend shows that IVDD predicts a probability greater than 0.8 for around half of the less druggable set. This points to a possible bias in the “less druggable” set. Indeed, a purely unsupervised approach such as this one, in which no *a priori* dichotomy is created, shows that several pockets are not judged to be less druggable. On the contrary, more than half are scored with high probability values. The less druggable nature can be ascribed partially to the shallow nature of this set; however, thanks to the large probe set to 3.5 Å, NanoShaper can still detect them.

This result hence partially contrasts with the *less druggable* labeling of this dataset. One should consider the principles behind this previous classification. Krasowski et al. (2011) postulated that a protein (not just the pocket) can be ascribed to the less druggable realm if none of the following conditions are met: 1) at least one ligand is orally available as judged by the Lipinski’s rule of five and 2) the ligands must have a clogP ≥–2 . In addition, the ligand efficiency of at least one of the ligands fulfilling criteria 1) and 2) must be ≥0.3 kcal mol^−1^ per heavy atom. To correctly fulfill the requirements one should be able to test all the chemical space before making any conclusion. Indeed, ideally, and more correctly, one could define the *true druggability* of a pocket as the value of the activity of the best possible ligand for that pocket in the chemical space. As the sampling of the chemical space is limited and further biases are due to the drug discovery community interest and efforts for a specific protein, this classification is questionable and not necessarily reliable. The problem of druggability classification of a pocket, or a protein, that is ligand-dependent is that it would require the true sampling of the chemical space. In our proposal, instead, we do not define *a priori* the labels but concentrate on the only reliable information that is, druggable pockets. The final result of this is that some pockets previously labeled as less druggable instead obtain high druggability probability values.

**FIGURE 7:**
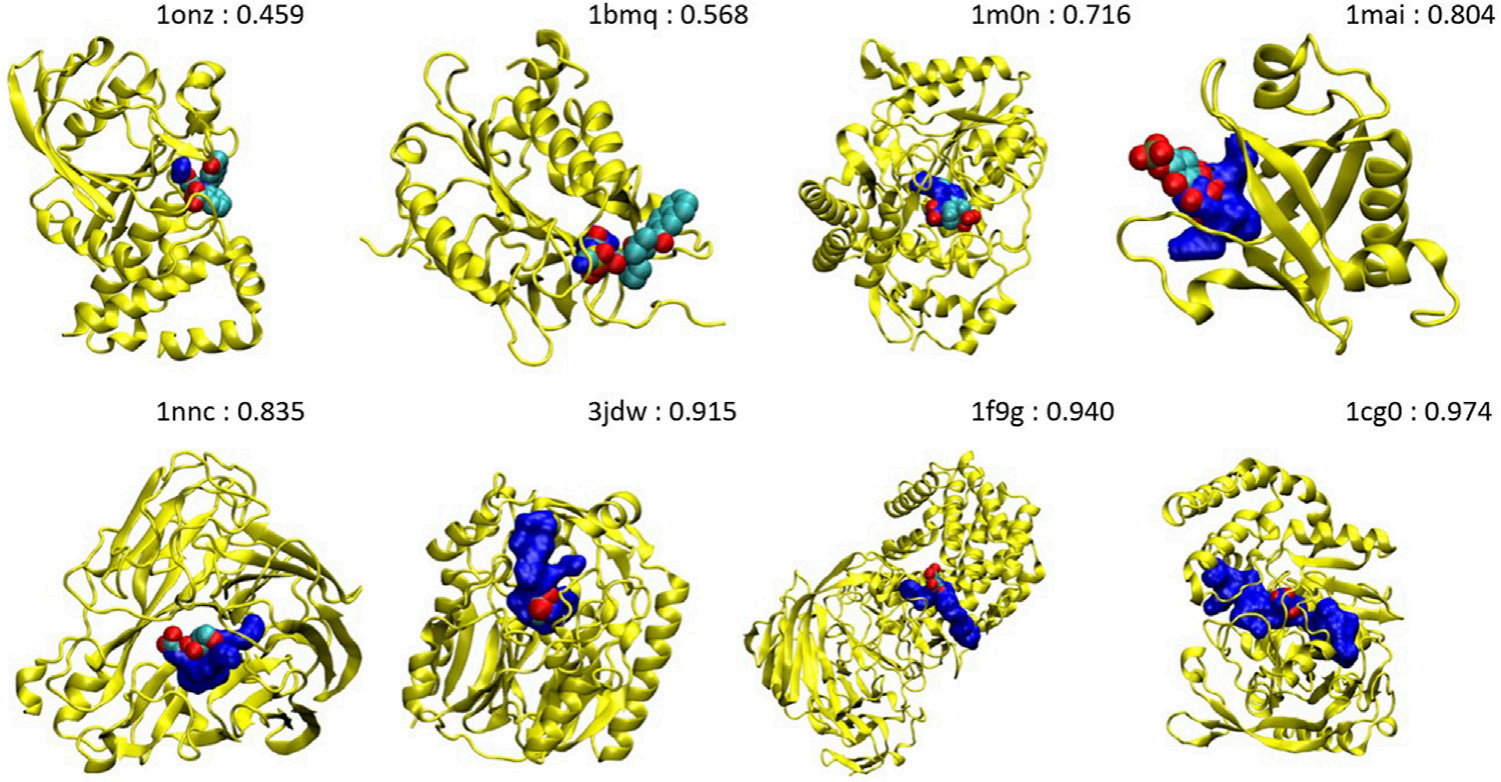
Main pockets (computed without hydrogen atoms) of *1onz*, *1bmq*, *1m0n*, *1mai*, *1nnc*, *3jdw*, *1f9g*, and *1cg0*. The pocket surface is in blue and the complexed ligand in the pdb file is in the VdW style. The number is the estimated druggability probability value.

It is interesting to analyze the probability shift from lower to upper values, systematically. Figure 7 shows the orthosteric pockets found by NanoShaper for the less druggable proteins, where we subsampled the structures set with a ratio of one every five complexes. The pockets here tend to become deeper and more compact moving from lesser probability to higher. The shift is particularly evident comparing *1onz* and *1cg0*, where the first case is a very shallow pocket, in which a ligand can be found, but it is neither a prototypical nor ideal pocket; its probability value is 0.46. In contrast, *1cg0* shows a much better defined and large enough pocket that would host a potential ligand well; IVDD classifies it as druggable with a probability value of 0.97. Except for *1qxo* (a pocket detected by NanoShaper that is too large), one can observe that the lower the score, the smaller and more shallow the pocket is. This is also evident looking at the portion of solvent-exposed surface of the ligands, where the low probability pockets tend to have more solvent-floating ligands.

There are some particularly interesting cases in this less druggable set, also considering the ligands found in the crystal structures. In *1kts*, *1gpu*, *1ucn*, and *1cg0* the ligands are small molecules or small molecule–like ligands. Missing these pockets would be quite negative in a drug discovery campaign. All these pockets score quite high with our method. One should not restrict to the pure small molecule paradigm; in the case where one is concerned with the design of a molecular glue or a PROTAC, even a warhead relatively not too active can be sufficient to degrade the protein. Our method is agnostic to ligand-induced labeling and avoids to miss or undervalue this kind of pockets.

**FIGURE 8:**
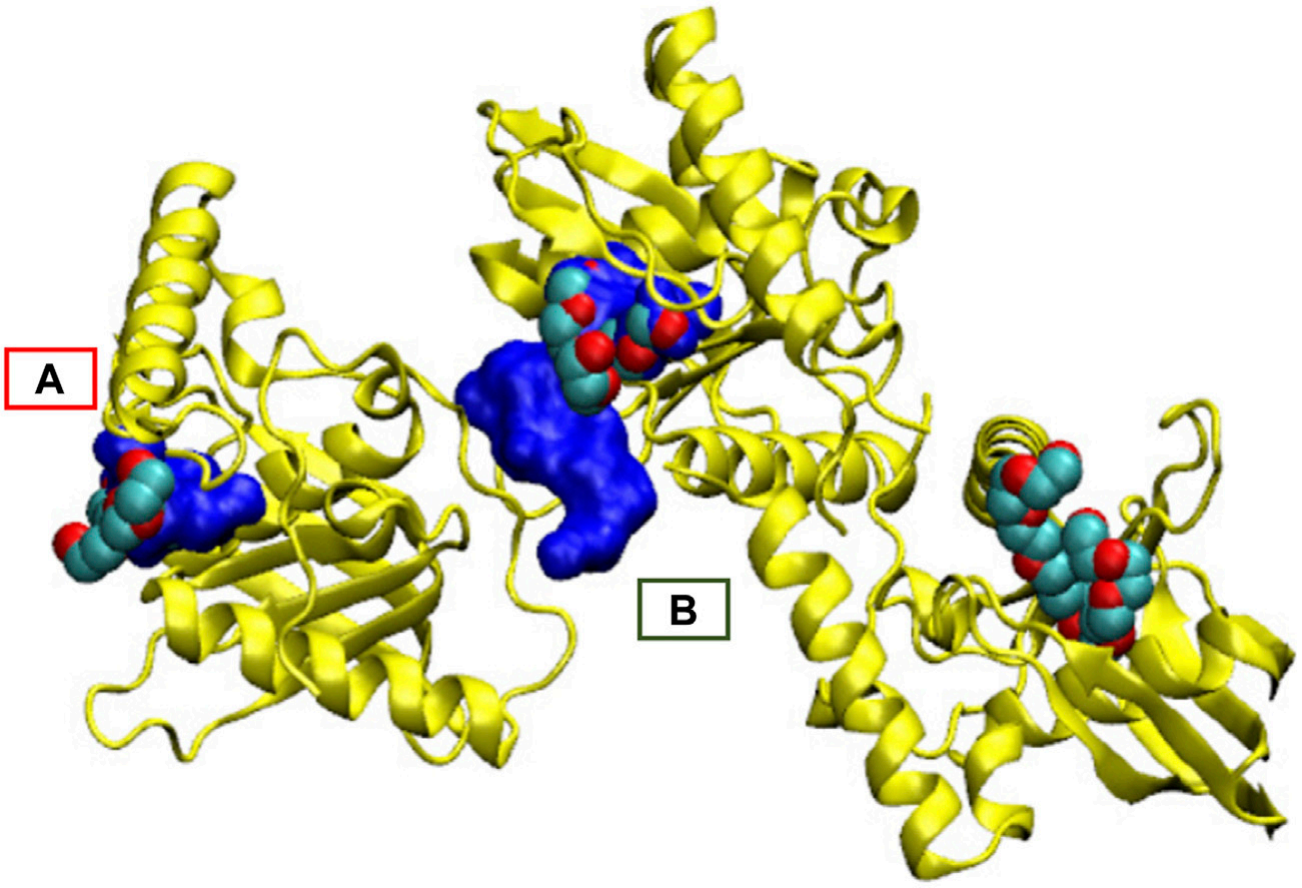
Main pocket shift for *1icj* together with the co-crystallized ligand. **(A)** Main pocket detected when adding hydrogen atoms. **(B)** Main pocket without adding hydrogen atoms. The pocket is semantically the same orthosteric pocket but changes from one monomer to another. The three structures of ligands bound in the PDB structure are also reported.

At a technical level, it is interesting to compare the pocket probabilities with and without hydrogen addition and to consider the NanoShaper's behaviour. As anticipated, adding or not adding hydrogen atoms does not change the detection of the main pocket by NanoShaper (highest Jaccard index). However, the shape and relative probability ranking both change. A first observation is that, in some peculiar cases, the percolating behavior of NanoShaper pockets cannot be solved by adding hydrogen atoms. Indeed, *1qxo* is still ranked last and, coherently, this pocket is percolating widely inside protein crevices. This global invariance is confirmed by analyzing *1icj* (see Figure 8). In this case, the detection of the main pocket is geometrically, but not semantically, changed when the structure with and without hydrogens atoms are considered. That is, the main detected pocket is the same but is in another monomer of the homotrimeric unit. Despite this finding, its druggability probability changes when adding hydrogen atoms. This demonstrates that the same pocket in two different conformations (monomers) is well-detected and always ranked as druggable. Indeed, without hydrogen atoms we can identify the orthosteric pocket in monomer A. Upon addition of hydrogen atoms, we instead identified the orthosteric pocket in monomer B. In this last case, the Jaccard index is higher with improved pocket quality (the pocket is more compact and located at the interface). However, the probability value changes as the corresponding geometry (and presence or absence of hydrogen atoms) changes, leading to a way higher value for pocket B. Therefore, from one side, what is judged druggable remains druggable. However, inside the druggable set, conformational changes of the same pocket have a non-trivial role in shifting the probability value. This confirms that it is crucial to consider dynamical aspects, particularly the probability of a given site conformation (and hence its free energy), in order to obtain a complete picture of the overall druggability of a site, which may be dealt with as a physical observable value.

Overall, this analysis shows that the dataset definition can create non-trivial biases, including biases due to labeling and the presence or absence of hydrogen atoms, which can induce local changes. One-class learning can mitigate the first bias because it only uses the druggable class during training. In the next section, we discuss other possible sources of bias and further evaluate the accuracy on a wider and curated dataset, also considering the initial processing of the structures (hydrogen addition).

### 3.4 PDTD Subset Validation

**FIGURE 9:**
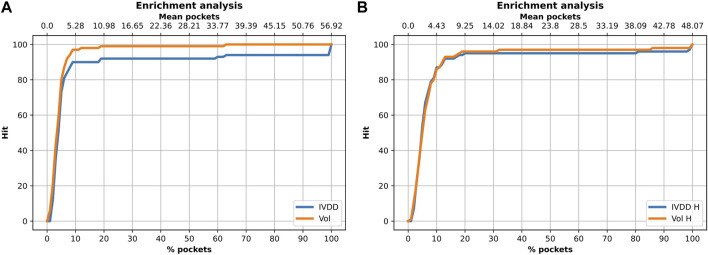
Enrichment analysis on the 100-protein experiment. **(A)** Solution without hydrogen atoms. For the 10% of pockets (for each protein) with the highest probability (on average 5.28 pockets), the orthosteric site is found in 90% of cases with IVDD and in 97% of cases with the descending ranking of the pocket volumes. **(B)** Solution with hydrogen atoms. For the 10% of the pockets (for each protein) with the highest probability (on average 4.43 pockets), the orthosteric site is found in 87% of cases with IVDD and in 86% of cases with the descending ranking of pocket volumes.

In this analysis, we used the 100-protein dataset, which is our curated subset of the PDTD. Here, we again evaluated the accuracy of classification and also searched for other possible sources of biases. It is well-known that the volume value has a crucial role in determining the druggability of a site. Among others, in Nayal and Honig (2006), the authors used SCREEN (Surface Cavity Recognition and Evaluation) to locate and analyze pockets in the NRDLD dataset. They observed that just picking the pocket with the highest volume value had a success rate of 64%. However, just looking at the volume value may create further biases, some intrinsic, some operational, and some technical. An overly large volume could be erroneously ascribed to the main site just because a small fraction contains the true binding site. This can happen in dependence of the pocket detection engine (e.g., for the percolation effect). Fortunately, this can be evaluated well *via* overlapping volume metrics or by the Jaccard index. Here, we performed this analysis by considering this issue. We compared our performance with that obtained by considering a simple descending ranking of the pocket volumes. Figure 9 and Table 2 show the results for the situations with and without hydrogen atoms. Using a simple ranking of the volume, we obtained a better performance at top 5, with an accuracy of 97%. This decreased to 89% when hydrogen atoms were added. In contrast, IVDD identified 90% of the orthosteric pockets in the top 5 highest probability pockets, which increased to 92% when hydrogen atoms were added. This shows that IVDD is more stable, although lower in accuracy in absolute terms.

**TABLE 2:** Results obtained on the PDTD subset (with and without hydrogen atoms) with the IVDD method and by a simple descending ranking of the pocket volumes. All results are referred to the orthosteric/main sites.

Description	IVDD	Volume	IVDD + H	Volume + H
Top 1	50	60	50	50
Top 2	67	76	69	65
Top 3	81	87	81	79
Top 5	89	97	92	89
Top 10%	90	97	87	86

**FIGURE 10:**
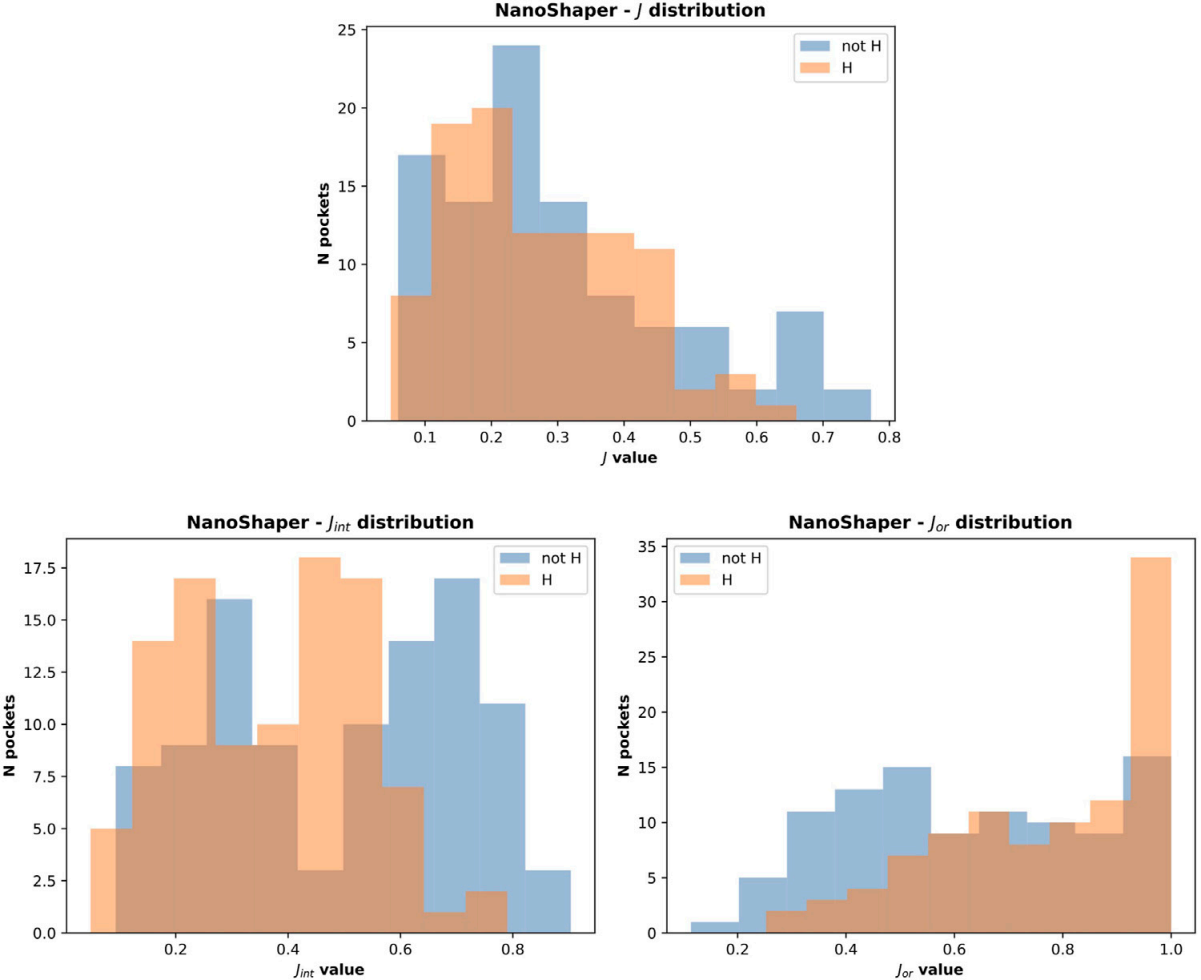
NanoShaper score distribution with and without hydrogen atoms.

**FIGURE 11:**
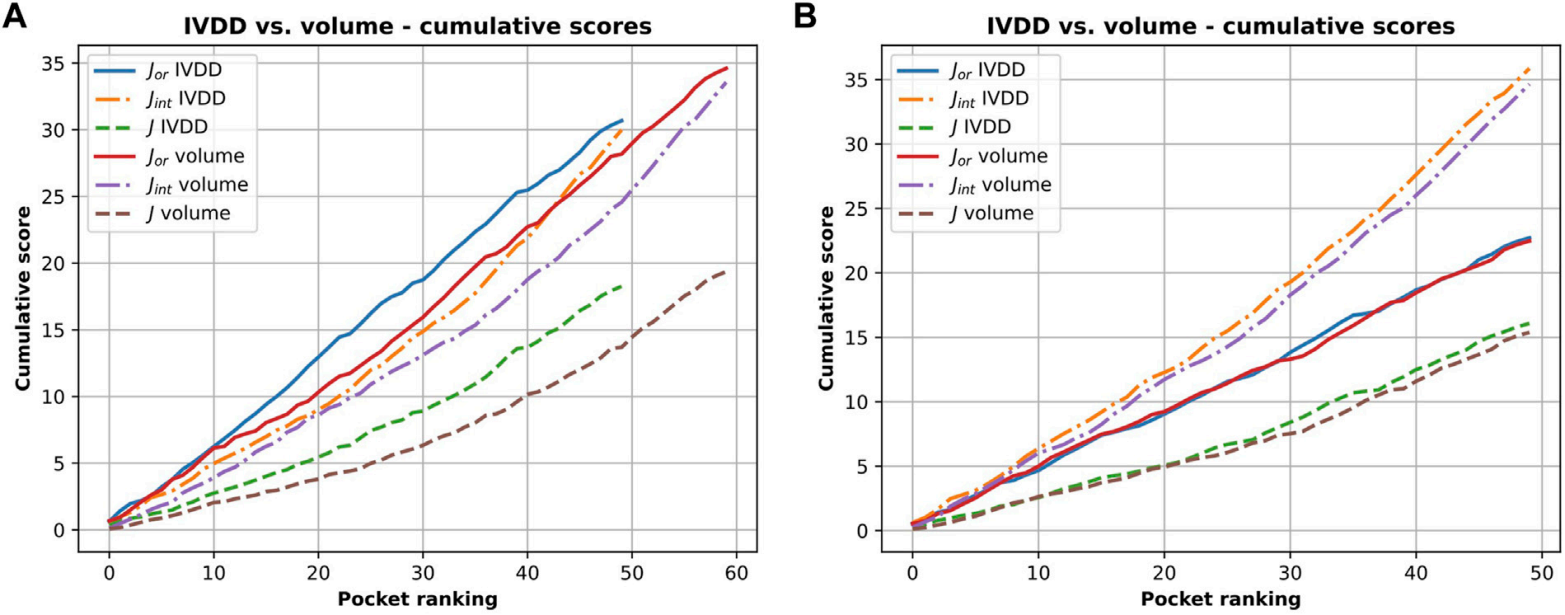
Cumulative scores (*J*, *J*_*or*_ and *J*_*int*_) for IVDD and volume ranking. Here, the orthosteric sites identified by IVDD and the volume ranking in top 1 are considered and ranked according to the probability score and the volume, respectively. Both the rankings are in descending order. Inset **(A)** is results without hydrogen atoms and inset **(B)** is with hydrogen atoms.

It is important to consider the quality of the pockets identified in both cases. The presence of hydrogen atoms sometimes allows the fragmentation of some of the overly large pockets. This not only increases the accuracy in terms of the main pocket druggability estimation but also affects the overall shape, which often becomes too tight. This is a NanoShaper-dependent effect, which is documented in Figures 10 and 11. In Figure 11, we reported the cumulative scores, namely *J*, *J*_*int*_, *J*_*or*_, for the volume and the IVDD ranking for the top 1 pockets, ordered respectively by volume and by probability. The trend shows a systematically higher value for all three scores for IVDD without hydrogen atoms and almost indistinguishable scores with hydrogen atoms. Interestingly, without hydrogen atoms, IVDD has a lower accuracy than that in the simple volume. This is unsurprising since an overly percolating volume allows easier main pocket detection. However, when quality is considered, even if some pockets are lost with IVDD, the remaining pockets have significantly higher scores. Again, we can mitigate a bias by not overfitting the volume-induced ranking. In the paradoxical case where one has a volume percolating throughout the protein, one would get a completely useless top 1 with 100% accuracy by using a pure volume ranking.

Within the IVDD results, it is also relevant to compare what happens with and without hydrogen atoms. Examining the structures that did not land in the top 5 positions with and without hydrogen atoms, one can conclude that most (e.g., *1vkg*, *1qpb*, and *1ht8*) are large pockets with low or intermediate Jaccard index or with very low *J*_*or*_ value. In some cases, there are shallow pockets (e.g., *1gp6* and *1i7g*) characterized by very high values of *J*_*or*_. Some of those structures improve in the presence of hydrogen atoms, reducing the number of targets that fall outside the top five from 11 to 8. Some shared structures (e.g., *1ht8*, *1h9u*, and *1v8b*) do not change the shape of the orthosteric pocket, leading to not significant changes in the probability.

We can compare the proposed solution to the many others in the literature. We have shown that by avoiding some of the possible biases (chiefly the labels) and considering the model without hydrogen atoms, we can obtain 81% detection accuracy in top 3 and 89% in top 5. We have also shown that a non-negligible fraction of the missed detections in top 5 can be ascribed to NanoShaper’s behavior. In comparison, Volkamer et al. (2012a) obtained 88% accuracy in correctly assigning to the druggable or non-druggable class in the NRDLD dataset with DoGSiteScorer, where the support vector machine is used as machine learning backend. In contrast, DrugPred (Krasowski et al., 2011) obtained 91% accuracy for NRDLD. A widely used method is fpocket from Le Guilloux et al. (2009), which correctly identified 83% of ligandable pockets in top 3 of all analyzed proteins. Overall, we achieved an accuracy that is similar to that of several existing methods but with some *ab initio* safeguards such as avoiding biases due to labels and volume.

**FIGURE 12:**
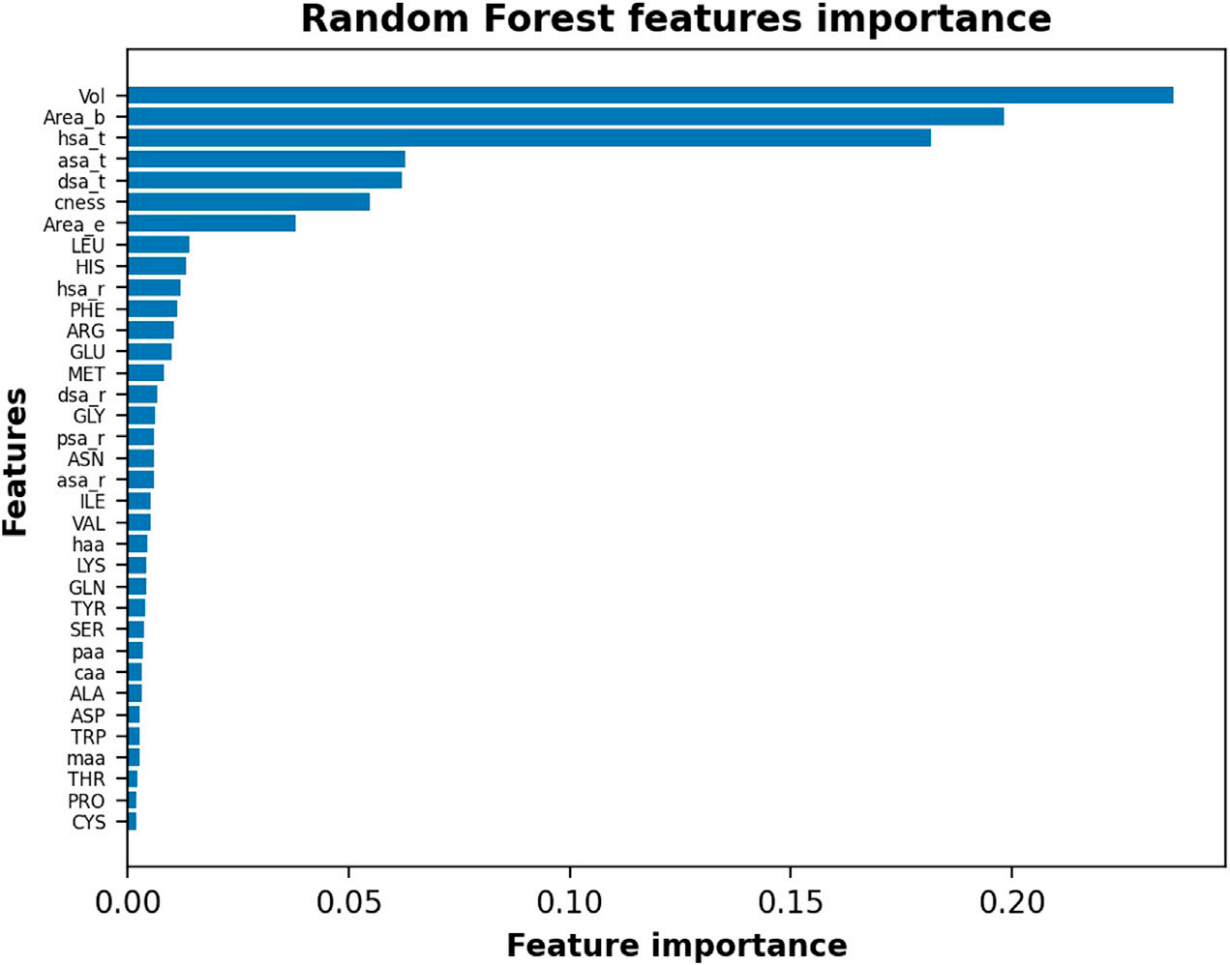
Random Forest features importance in the descending order by assigning *ex post* labels to the IVDD predictions. Results shown are without hydrogen atoms; similar results are obtained with hydrogen atoms.

To further investigate the IVDD results, we identified how much each single feature affects the IVDD prediction. IVDD does not embed a feature selection method, so we used an *ex post* labeling strategy. We first estimated the probability obtained, on average, for each orthosteric site in the dataset, obtaining 0.852 and 0.877, respectively, without and with hydrogen atoms. These values represent two thresholds and allow a labeling for each binding site, which is 0 when its probability is lower than the threshold value, otherwise 1. This *ex post* labeling allows us to fit a classifier (here, we chose a random forest classifier (Breiman, 2001) with 100 estimators and the Gini index as criteria for the split) and to estimate the features importance. Figure 12 shows the results of this additional experiment. Volume (Vol) is a major impacting feature, followed by area of the pocket surface (Area_b), hydrophobic surface area, hydrogen-bond acceptor surface area (asa_t), hydrogen-bond donor surface area (dsa_t), binding site compactness (cness), and entrance (mouth) surface area (Area_e). Similar results can be obtained with different classifiers and can be found in Supplementary Material Section S3.

To further check these results, we ran this experiment by normalizing data. We found that hydrophobic surface, polar surface, and volume still dominate the model. This means that IVDD is influenced by the volume, but it also considers other chemical aspects in predicting probability. Of less relevance is the fact that hydrophobic residues (LEU, PHE, MET, and GLY) and some charged residues (HIS, GLU) rank slightly higher. The presence of hydrophobic residues and volume as key factors is largely consistent with chemical intuition.

**FIGURE 13:**
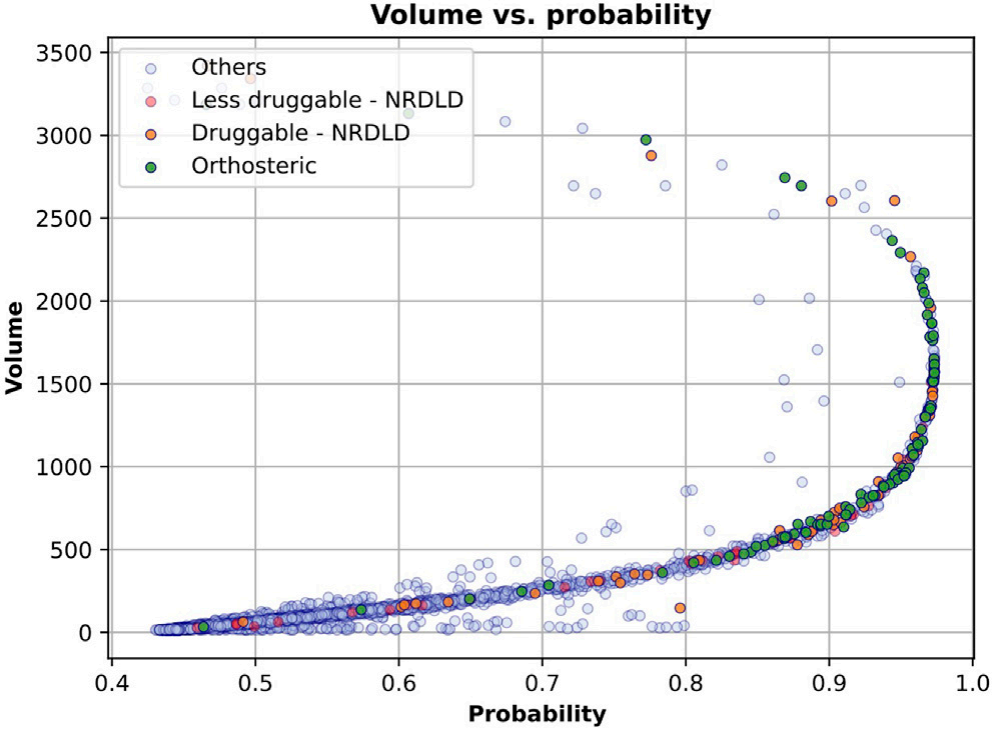
IVDD probability scores vs. volume. Each sample represents a pocket (colored according to the corresponding dataset). The *x*-axis represents the probability that a pocket is druggable, while the *y*-axis represents the volume of each pocket. The plot is referred to the solution with hydrogen atoms. Similar results are obtained without hydrogen atoms.

The correlation between IVDD prediction and volume can be seen in Figure 13, in which we have plotted each binding site as a point in the 2D space, where the coordinates are the probability predicted by IVDD and the volume itself. In the presence (see Figure 13) and absence (data not shown) of hydrogen atoms, the samples with the highest probability have a volume between 500 and 2,000 Å^3^. The orthosteric sites and the training samples are condensed on the right side of the figure, meaning that they obtained high probability scores in most cases. Non-orthosteric binding sites are condensed in the bottom left of the figure since they are mostly small pockets and obtain low probability scores. However, both figures contain some non-orthosteric pockets with a volume between 1,000 and 2,000 Å^3^ and lower probability scores. In such cases, the IVDD decision has been influenced by factors other than volume.

## 4 Discussion and Conclusions

In this study, we presented an unsupervised one-class approach to build a druggability estimation model. We defined a pipeline to obtain all the pockets of a protein (NanoShaper), their corresponding descriptors, and druggability prediction. The method achieved 89% accuracy in top 5, in line with other methods. Although the method was less accurate than a trivial volume-based ranking by NanoShaper, it favors well-shaped pockets with higher *J*, *J*_*or*_, and *J*_*int*_ scores. This has practical relevance since a relatively tight and well-shaped pocket reduces the ambiguity and difficulty of the subsequent virtual screening and docking campaigns. Crucially, the proposed method does not aim to distinguish between druggable and less druggable pockets (binary classification). Rather, a probability for pocket is given, which is easily interpretable and comparable across different proteins. In contrast to a score, the probability estimation does not need *a posteriori* calibration. Rather, the logistic model of the hypersphere naturally delivers this information. Again, a probability allows the computational medicinal chemist to easily identify the most eligible pocket for subsequent drug discovery steps, without wondering if the score value is high or low in absolute terms. This is because any probability very close to one is inevitably a strong indicator. Most importantly, this approach does not need to define a less druggable or non-druggable class. This potentially ambiguous concept is bypassed by the one-class approach. The results show that druggability prediction is best considered as a concept learning problem, rather than a classification problem. This approach allows de-biasing from the start of the learning process, which is clear in the results from the less druggable dataset. We also found that the presence or absence of hydrogen atoms can change the overall modeling attempt in ways that are not always obvious. This is because the effects of NanoShaper are overimposed on the IVDD learning model. Our proposal to mitigate and reduce various biases, even at the cost of lower accuracy, is indebted to the fair machine learning field (Jiang and Nachum, 2019). While fairness concepts are usually applied to social aspects (e.g., demographic parity), we draw on this way of thinking to focus on certain label information only.

Together with explicit structural biases, technical aspects also have an important role. We tested several different values for the small and large NanoShaper probes (data not shown) to identify the pockets. The small probe was easy because there is no reason not to choose the water molecule–like size of 1.4 Å. For the large probe, there is no immediate physically driven quantity, with the convex hull being the extreme solution. We found that a value of 3.5 Å performed better than 3 Å in detecting relatively shallow pockets together with the more prototypical buried ones. Larger values generally led to poorer results in terms of shape, with a systematic decrease in Jaccard index values.

In terms of future developments, we envision several improvements of our methodology. A volume segmentation *ad hoc* algorithm could improve the accuracy, particularly when selecting the value of the large probe. Such a tool could provide more freedom of choice for this parameter. The work of Aggarwal et al. (2021), among others, has shown that many pieces of software for pocket identification tend to identify large pockets without segmentation techniques. Segmentation could be used to find subpockets that are better suited to virtual screening and docking. Another development would be a web server to easily access the tool. Finally, we plan to combine this method with the Pocketron method (La Sala et al., 2017) to not only track the pocket volume and residues over time but also to provide a dynamic druggability score that explicitly considers the probability of the conformation ultimately delivering a Boltzmann weighted estimator.

## Data Availability

The original contributions presented in the study are included in the article/Supplementary Material, further inquiries can be directed to the corresponding author.
