# Supplementary material for: Probabilistic Pocket Druggability Prediction via One-Class Learning
Source: Front Pharmacol. 2022 Jun 29;13:870479. doi: 10.3389/fphar.2022.870479 (PMC9278401; doi:10.3389/fphar.2022.870479)
Supplement: Supplementary file 1 [file DataSheet1.PDF]

Supplementary Material for Probabilistic pocket druggability prediction via one-class learning

1 PROTEINS DESCRIPTION: NRDLD DATASET

Table S1 describes all the proteins included in the druggable (training) and the less druggable datasets. Druggable proteins are marked with d (training set), less druggable proteins are marked with n.

PDB code	Name	Category
1pwm	Aldose reductase	d
1lox	15-lipoxygenase	d
3etr	Xanthine oxidase	d
3f1q	Dihydroorotate dehydrogenase	d
3ia4	Dihydrofolate reductase	d
2cl5	Catechol-O-methyltransferase	d
1uou	Human thymidine phosphorylase	d
1t46	c-Kit kinase	d
1unl	cyclin-dependent kinase5	d
1q41	Glycogen synthase kinase 3	d
2i1m	FMS kinase	d
1pmn	c-Jun kinases	d
1fk9	HIV reverse transcriptase (nonnucleoside reverse transcriptase inhibitor binding site)	d
1e66	Acetylcholinesterase	d
1xoz	Phosphodiesterase 5A	d
1owe	Urokinase plasminogen activator	d
1r55	A disintegrin and metalloprotease	d
3f0r	Histone Deacetylase 8	d
1oq5	Carbonic anhydrase II	d
1kzn	DNA gyrase	d
2aa2	Mineralocorticoid receptor	d

3b68	Androgen receptor	d
1sqn	Progesterone receptor	d
1v16	Branched-chain alpha-keto acid dehydrogenase	n
3jdw	Arginine:glycine amidinotransferase	n
1ajs	Aspartate aminotransferase	n
1wvc	CDP-D-glucose synthase	n
1kc7	Pyruvate phosphate dikinase	n
1mai	Phospholipase C	n
1px4	Beta-galactosidase	n
1od8	Xylanase	n
1bmq	Interleukin-1 beta-converting enzyme 1	n
1bls	Beta-lactamase	n
1m0n	Dialkylglycine Decarboxylase	n
1ec9	D-glucarate dehydratase	n
1b74	Glutamate racemase	n
1g98	Phosphoglucose isomerase	n
1e9x	Cytochrome P450 14alpha -sterol demethylase	d
1hw8	3-hydroxy-3-methylglutaryl-CoA	d
1sqi	4-hydroxyphenylpyruvate dioxygenase	d
1r9o	Cytochrome P450 2C9	d
4cox	Cyclooxygenase 2	d
1c14	Enoyl reductase	d
2bxr	Monoamine oxidase A	d
2gh5	Glutathione reductase	d
1hvy	Thymidylate synthase	d
1rsz	Purine nucleoside phosphorylase	d
1n2v	tRNA-guanine transglycosylase	d
1v4s	Hexokinase	d
1u4d	ACK1 kinase	d

1m17	Epidermal growth factor receptor kinase	d
2dq7	Fyn kinase	d
1qpe	Lck kinase	d
1qhi	Thymidine kinase	d
2fb8	B-Raf kinase	d
1ke6	cyclin-dependent kinase2	d
2br1	Chk1 kinase	d
1ywr	p38 Mitogen-activated protein kinases	d
2ivu	RET kinase	d
2hiw	Abl tyrosin kinase	d
2i0e	Protein kinase C	d
1ywn	Vascular endothelial growth factor receptor-2	d
1ig3	Thiamin pyrophosphokinase	d
1yvf	Hepatitis C virus polymerase NS5B	d
1k8q	Gastric lipase	d
1kvo	Phospholipase A 2	d
1xm6	Phosphodiesterase 4B	d
1udt	Phosphodiesterase 5	d
1u30	Amylase	d
1r58	Methionine aminopeptidase-2	d
1rwq	Dipeptidyl peptidase-IV	d
1lpz	Factor Xa	d
2g24	Renin	d
1hvr	HIV protease	d
1gkc	Matrix metalloproteinase-9	d
1yqy	Lethal factor	d
1o5r	Adenosine deaminase	d
1js3	DOPA decarboxylase	d
1k7f	Tryptophan synthase	d

1j4i	FKBP13	d
1vbm	Tyrosyl-tRNA synthetase	d
1rv1	Ubiquitin-protein ligase E3 Mdm2	d
1gwr	Estrogen receptor	d
1m2z	Glucocorticoid receptor	d
3d4s	Beta-2-adrenergic receptor	d
1ai2	Isocitrate dehydrogenase	n
3pcm	3,4-dioxygenas	n
1d09	Aspartate transcarbamoylase	n
1c9y	Ornithine carbamoyltransferase	n
1gpu	Transketolase	n
1qmf	Penicillin binding protein-2X	n
1moq	Glucosamine 6-phosphate synthas	n
1ucn	Nucleoside diphosphate kinase	n
1t03	HIV reverse transcriptase (nucleoside binding site)	n
1qs4	HIV integrase	n
1fth	Acyl carrier protein synthase	n
1rnt	Ribonuclease T2	n
1onz	Protein-tyrosine phosphatase 1B	n
1x9d	Mannosidase	n
1nnc	Neuraminidase	n
1olq	Endo-beta-1,4-glucanase	n
1jak	Beta-N-Acetylhexosaminidases	n
1kts	Thrombin	n
1nlj	Cathepsin K	n
1icj	Peptide deformylase	n
1hqg	Arginase	n
2gsu	Phosphodiesterase-nucleotide Pyrophosphatase	n

1g7v	3-deoxy-D-manno-2-octulosonate-8-phosphate synthase	n
1f9g	Hyaluronate lyase	n
1qxo	Chorismate synthase	n
2gyi	D-xylose isomerase	n
1o8b	Ribose-5-phosphate isomerase	n
1cg0	Adenylosuccinate synthetase	n

Table S1: Proteins description of the NRDLD dataset.

2 PROTEINS DESCRIPTION: PDTD DATASET

Table S2 describes all the proteins included in the PDTD (100-proteins) dataset.

**PDB code**	**Name**	**Category**
1a28	Progesterone receptor	d
1acj	Acetylcholine esterase	d
1aco	Aconite with transaconitate bound	d
1adc	NAD analogues bound to alcohol dehydrogenase	d
1coy	Cholesterol oxidases	d
1cqe	Prostaglandin H2 synthase-1	d
1d3g	Dihydroorotate dehydrogenase	d
1d6u	E. Coli amine oxidase	d
1db1	Nuclear receptor for vitamin D	d
1dht	Estrogenic 17-beta hydroxysteroid dehydrogenase	d
1diy	Cyclooxygenase active site of PGHS-1	d
1dkf	Heterodimeric complex of RAR and RXR	d
1e1f	Beta-glucosidase	d
1e3g	Androgen receptor	d
1e3k	Progesteron receptor	d
1e55	Mutant Monocut beta-glucosidase	d
1eet	HIV-1 reverse transcriptase	d

1efh	Hydroxysteroid sulfotransferase	d
1f2a	Cruzain hydrolase	d
1fm6	Heterodimer of the RXR-α and PPAR-γ	d
1gii	Cyclin dependent kinase	d
1gos	Monoamine oxidase B	d
1gp6	Anthocyanidin synthase	d
1gpk	Acetylcholinesterase	d
1gqs	Acetylcholinesterase complexed with NAP	d
1gs4	Androgen receptor ARccr	d
1h5u	Glycogen phosphorylase B	d
1h9u	Retinoid X receptor beta	d
1hb2	Isopenicillin N synthase	d
1hdy	Alcohol dehydrogenase variant	d
1hfc	Fibroblast collagenase	d
1hj1	Estrogen receptor beta	d
1hld	Liver alcohol dehydrogenase	d
1ho4	Pyridoxine 5-phosphate	d
1ht8	Oxidoreductase COX-1	d
1hy3	Estrogen sulfotransferase V269E	d
1hzx	Bovine Rhodopsin	d
1i7g	Human PPAR-α	d
1ie9	Nuclear receptor for vitamin D	d
1iiu	Plasma retinol-binding protein	d
1j90	Deoxyribonuclease kinase	d
1jbp	Catalytic subunit of c-AMP dependent protein kinase	d
1jkh	HIV-1 reverse transcriptase	d
1js3	Dopa decarboxylase	d
1k3u	Tryptophan synthase	d
1k4w	Nuclear receptor ROR-	d

1k74	Heterodimer of PPAR- and RXR-	d
1k7l	Human PPAR-	d
1lde	Liver alcohol dehydrogenase	d
1ldy	Liver alcohol dehydrogenase complexed to NADH and cyclohexyl formamide	d
1mup	Pheromone binding to two urinary proteins	d
1n7i	Phenylethanolamine N-metyltransferase	d
1nwk	Monomeric actin in the ATP state	d
1og5	Human cytochrome P450 CYP2C9	d
1oi9	Human thr160-phospho CDK2/cyclin A	d
1p1n	GluR2 ligand binding core (S1S2J) mutant	d
1p2d	Glycogen phosphorylase B	d
1p4g	Glycogen phosphorylase B in complex with C-(1-azido-alpha-D-glucopyranosyl) formamide	d
1p93	Glucocorticoid receptor	d
1pcg	Helix-stabilized cyclic peptides	d
1pha	Cytochrome P450-CAM	d
1pig	Pancreatic alpha-amylase	d
1ppl	Aspartyl proteinases	d
1qab	Retinol binding protein	d
1kvo	Phospholipase A 2	d
1qkm	Estrogen receptor β	d
1qkt	Mutant estrogen nuclear receptor	d
1qpb	Pyruvate decarboxylase	d
1r18	Isoaspartyl methyltransferase	d
1r1k	Heterodimer EcR/USP bound to ponasterone A	d
1rbp	Serum retinol binding protein	d
1rlb	Retinol binding protein complexed with transthyretin	d
1rt6	HIV-1 reverse transcriptase	d

1tvr	HIV-1 RT/9-CL TIBO	d
1uhl	LXRα-RXRβ LBD heterodimer	d
1ulb	Purine nucleoside phosphorylase	d
1uom	Estrogen receptor complexed with Tetrahydroisochiolin	d
1upv	Liver X receptor β	d
1v8b	Hydrolase	d
1vkg	HDAC8	d
1vlb	Aldehyde oxidoreductase	d
1vot	Acetylcholine esterase	d
1w6k	Human OSC	d
1x07	Undecaprenyl pyrophosphate synthase	d
1xnx	Androstane receptor	d
1y0s	PPAR-γ	d
1zhy	Oxysterol binding protein Osh4	d
2a3i	Mineralocorticoid receptor	d
2a3l	Adenosine 5'-Monophosphate deaminase	d
2ack	Acetylcholinesterase	d
2ae2	Tropinone reductase-II	d
2bx8	Human serum albumin	d
2dln	D-alanine ligase	d
2mas	Purine nucleoside hydrolase	d
3bto	Liver alcohol dehydrogenase	d
3ert	Estrogen receptor-α	d
3hvt	Human immunodeficiency virus type 1 reverse transcriptase heterodimer	d
4thi	Thiaminase I	d
6cox	Cyclooxygenase-2	d
8cat	Liver catalase	d

Table S2: Proteins description of the PDTD dataset.

3 FEATURES IMPORTANCE - RESULTS FROM DIFFERENT CLASSIFIERS

In this section we tested the robustness of the features importance previously obtained by the Random Forest classifier.

Two additional classifiers are fitted, both based on trees: the Decision tree classifier and the AdaBoost classifier. We adopted tree-based classifiers since they can provide features importance without requiring any feature normalization step (regression based methods require normalization before usage). In all the cases, we used the scikit-learn library (Pedregosa et al., 2011), with all the default parameters. Figure S1 shows the results of this additional experiment. In all the cases, the volume (Vol) is the major impacting feature. Additionally, the area of the pocket surface (Area_b), the hydrophobic surface area (hsa_t), the binding site compactness (cness) have a high impact in all the cases.

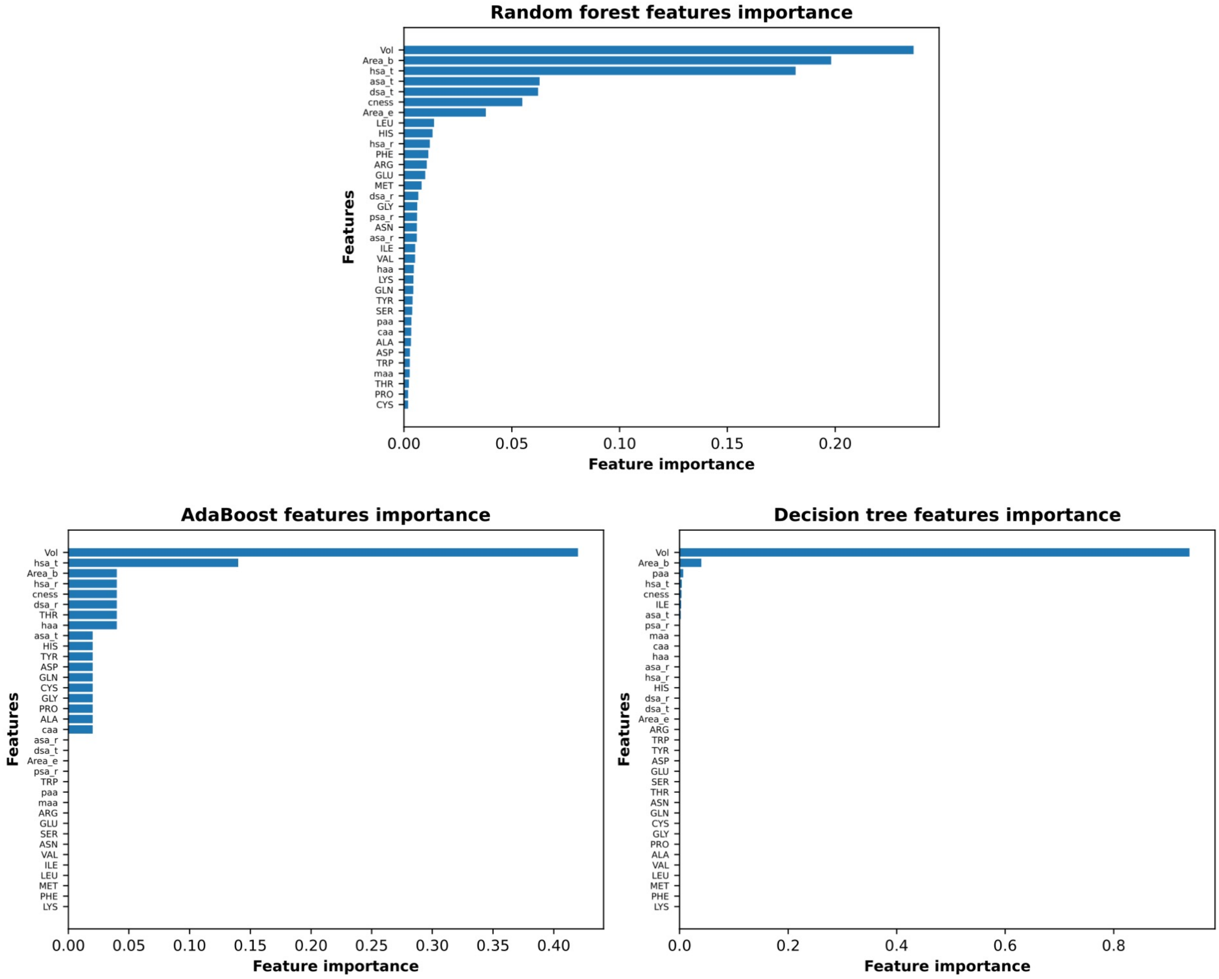

Figure S1. Features importance by assigning ex post labels to the IVDD predictions. Results shown are without hydrogens; similar results are obtained with hydrogens.

REFERENCES

Pedregosa, F., Varoquaux, G., Gramfort, A., Michel, V., Thirion, B., Grisel, O., et al. (2011). Scikit-learn: Machine learning in Python. *Journal of Machine Learning Research* 12, 2825–2830
